# Glucagon-Like Peptide-1 Receptor Agonists in the Treatment of Idiopathic Inflammatory Myopathy: From Mechanisms of Action to Clinical Applications

**DOI:** 10.7759/cureus.51352

**Published:** 2023-12-30

**Authors:** Shilpa Rajagopal, Faisal Alruwaili, Vasilis Mavratsas, Myrna K Serna, Vijaya L Murthy, Mukaila Raji

**Affiliations:** 1 John Sealy School of Medicine, University of Texas Medical Branch, Galveston, USA; 2 Hospital Medicine, Stormont Vail Health, Topeka, USA; 3 Department of Internal Medicine and Aerospace Medicine, University of Texas Medical Branch, Galveston, USA; 4 Division of General Medicine, Department of Internal Medicine, University of Texas Medical Branch, Galveston, USA; 5 Division of Rheumatology, Department of Internal Medicine, University of Texas Medical Branch, Galveston, USA; 6 Division of Geriatrics and Palliative Medicine, Department of Internal Medicine; Department of Preventive Medicine and Population Health, University of Texas Medical Branch, Galveston, USA

**Keywords:** glp-1 receptor agonist, inflammatory myositis, muscle atrophy, muscle weakness, idiopathic inflammatory myopathy

## Abstract

Idiopathic inflammatory myopathies (IIMs) result in proximal muscle weakness and other intramuscular and extramuscular manifestations. Pharmacologic treatments in use for IIMs are limited to corticosteroids and immunosuppressants in addition to supportive physical and occupational therapy. Glucagon-like peptide-1 receptor (GLP-1R) agonists are currently utilized in the treatment of type II diabetes and obesity but may play a role in the treatment of IIMs. The current scoping review of extant literature aims to synthesize findings from studies assessing the therapeutic effects of GLP-1R agonists in the management of inflammatory myopathy and muscle atrophy. A literature search was conducted through PubMed, resulting in a total of 19 research-based articles included in this review. Mice and human studies showed, with varying levels of significance, that GLP-1R agonists led to decreases in muscle atrophy, inflammation, adiposity, and weakness; improvement in muscle microvasculature and endurance; and promotion of muscle mitochondria biogenesis. The potential for GLP-1R agonists to improve muscle function and architecture underscores the need for large randomized controlled, clinically comparative trials of GLP-1R agonists in patients with IIM.

## Introduction and background

Idiopathic inflammatory myopathies (IIMs), which encompass such conditions as polymyositis (PM), dermatomyositis (DM), and inclusion body myositis (IBM), are rare diseases that involve a multifactorial autoimmune pathophysiology impacting various organ systems [1-3]. In the United States, PM and DM affect about 5 to 22 per 100,000 people, with higher rates in women, older adults, and Black individuals [4]. The primary clinical presentation for IIM is proximal muscle weakness and can be accompanied by extra muscular manifestations, including rashes, dysphagia, and cardiac and pulmonary complications [2]. Laboratory testing for muscle enzymes and myositis-specific autoantibodies, electromyography, and muscle biopsy are common techniques used for the diagnosis of IIM [5].

Current treatment for IIM includes both pharmacological and supportive elements to help decrease the severity of symptoms. Corticosteroids are often the first-line agents used to decrease inflammation; immunosuppressants are also prescribed in refractory IIM cases and to help taper long-term corticosteroid use [6-9]. Furthermore, patients with IIM, especially those with IBM [10], tend to experience limited mobility with disease progression, hindering their ability to carry out activities of daily living and leading to reduced quality of life [11]. Accordingly, many individuals living with IIM tend to rely on assistive devices and receive physical and occupational therapy services.

Given the small number of medication-based treatments currently available for IIM management, there is a need to identify new lines of therapy. In September 2021, the Food and Drug Administration (FDA) granted Orphan Drug status, a label reserved for rare diseases, to PF1801, a glucagon-like peptide-1 receptor (GLP-1R) agonist, for the treatment of PM [12]. Previously, the FDA provided PF1801 with an Orphan Drug designation for Duchenne muscular dystrophy (DMD) based on the drug’s observed impact on decreasing muscle atrophy [13,14].

Mechanistically, GLP-1R agonists target and activate GLP-1R, leading to a glucose-dependent increase in insulin secretion and feelings of satiety [15]. They have also been associated with reduced expression and release of proinflammatory cytokines [16]. Thus, while GLP-1R agonists are commonly used for type II diabetes and obesity, there is a growing body of evidence about their role in mediating the effects of other conditions involving inflammatory pathways, including asthma, atherosclerosis, neurogenerative disorders, and myopathies [17,18]. Yet, GLP-1R agonists possess multiple side effects, many of which are gastrointestinal; some documented contraindications for GLP-1R include severe renal and gastrointestinal diseases, thyroid and endocrine malignancies, and pregnancy [19].

Studies about the therapeutic effects of GLP-1R agonists in the management of inflammatory myopathy and muscle atrophy are relatively limited, in part due to the research constraints of examining clinically rare diseases like IIM. This scoping review article seeks to synthesize current findings related to GLP-1R agonism and inflammatory myopathy, with the goal of guiding future evidence-based management of IIM.

Sections of this review were previously presented as a poster at the American Geriatrics Society 2023 Annual Scientific Meeting in May 2023 [1].

## Review

Methods

A scoping literature review of research studies published from 2010 to December 2023 was conducted through PubMed using the following medical subject heading (MeSH) search terms: (glucagon-like peptide-1 AND Inflammatory Myopathy) OR (glucagon-like peptide-1 AND Skeletal Muscle). The initial search yielded 194 articles, of which 34 were excluded based on their classification as review-type articles. Following an assessment of the article titles, keywords, and abstracts, a total of 19 studies were selected for final analysis based on subject matter relevance on the role of GLP-1R agonists on muscle weakness and atrophy. Studies were deemed to meet the inclusion criteria if they discussed GLP-1 in the context of skeletal muscle, were research studies, were peer-reviewed, were written in English, and were published between the designated dates. Criteria for exclusion included inapplicability to the search term topics, lack of full-text availability, review articles, editorials, and non-English articles. The following information was extracted from the selected articles: methods and study design model, research subjects, measured outcomes, and main findings from the results and discussion.

Results

Myogenic and Anti-inflammatory Properties

GLP-1 agonists have been shown to suppress the expression of muscle atrophic factors and promote the effect of myogenic factors. The expression of myostatin and muscle atrophic factors, such as the F-box only protein 32 (atrogin-1) and muscle RING-finger protein-1 (MuRF-1), decreased following treatment with a GLP-1 agonist in aged mice and dexamethasone-treated C2C12 myotubes, a type of mouse skeletal muscle cell line [20,21]. Conversely, myogenic factors, such as the myoblast determination protein 1, were found to have increased with GLP-1 agonism [20,21].

GLP-1 agonists have also demonstrated several anti-inflammatory effects. The levels of inflammatory cytokines interleukin-6 and tumor necrosis factor-alpha were decreased following the treatment of aging mice with a GLP-1 agonist, possibly through the regulation of OPA-1-TLR-9, which mediates inflammatory responses [21].

Mitochondrial Preservation and Muscle Microvasculature Effects

Mitochondrial function is central to the metabolism of skeletal muscle. Studies found that GLP-1 agonism promotes mitochondrial biogenesis and increases mitochondrial content in animal models [22,23]. This shift to a more oxidative metabolism may be related to the skeletal remodeling that has also been shown to occur. Additionally, GLP-1 agonism may cause a shift to a greater proportion of high-endurance Type I and Type IIa muscle fibers [21,23]. Various animal models of muscle atrophy, including chronic kidney disease (CKD)-induced atrophy, DMD, and steroid-induced atrophy, undergo amelioration of this atrophy [20,24].

GLP-1 agonism has been shown to enhance muscle protein synthesis in humans [25]. The use of GLP-1 agonists for the treatment of obesity is rapidly becoming more widespread. Notably, this effect appears to mainly involve a reduction in the mass of adipose tissue with relative preservation of muscle mass [26]. The increase seen in high-endurance muscle fibers appears to correspond with a functional increase in endurance. Overexpression of GLP-1 in the gastrocnemius muscle of mice increased their exercise endurance [23]. In a mouse model of CKD, treatment with a GLP-1 agonist improved grip strength [20].

Moreover, microvascular blood flow and volume were found to increase with increasing GLP-1 agonism [27]. One study found that GLP-1 infusion acutely increased muscle microvascular blood volume within 30 minutes without altering microvascular blood flow velocity [28]. This effect persisted throughout the 150-minute infusion period, contributing to a significant increase in muscle microvascular blood flow [28].

**Figure 1:**
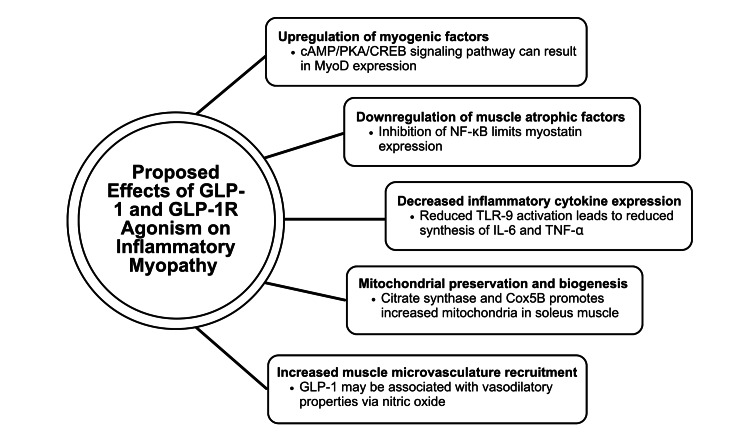
Documented effects of GLP-1 and GLP-1R agonists on muscle atrophy and myopathy GLP-1: Glucagon-like peptide-1; GLP-1R: Glucagon-like peptide-1 receptor

Primary findings from the 19 research studies are included in Table 1. The suggested mechanisms by which GLP-1 and GLP-1R agonism affect inflammatory myopathies are summarized in Figure 1.

**Table 1:** Summary of articles discussing GLP-1 agonism in relation to inflammatory myopathy, muscle weakness, and muscle atrophy

Study	Article Type	Key Findings
Wang et al., 2011 [29]	Bench study (mice)	GLP-1 agonism through Exendin-9 promotes myotubular differentiation.
Chai et al., 2012 [30]; Dong et al., 2013 [31]	Bench study (mice)	GLP-1 agonism recruits muscle microvasculature via a nitric oxide-dependent pathway.
Hong et al., 2019 [20]	Bench study (mice)	GLP‐1R agonists ameliorate muscle wasting by suppressing muscle atrophic factors and enhancing myogenic factors through GLP‐1R‐mediated signaling pathways.
Gurjar et al., 2020 [24]	Bench study (mice)	Liraglutide restores myofibrillar architecture in muscle atrophy.
Xu et al., 2020 [32]	Bench study (mice)	GLP-1 agonism through exenatide ameliorates intramyocellular lipid deposition.
Khin et al., 2021 [21]	Bench study (mice)	Dulaglutide increases myofibers and improves muscle function by attenuating inflammation.
Wu et al., 2022 [23]	Bench study (mice)	Exercise-induced GLP-1 release by skeletal muscle and GLP-1 improves skeletal muscle endurance capacity.
Yamada et al., 2022 [22]	Bench study (mice)	Liraglutide preserves mitochondria in muscle in T2DM mice.
Kamiya, Mizoguchi, & Yasuda, 2022 [33]	Bench study (humans + mice)	Muscle biopsies of dermatomyositis and polymyositis patients demonstrate the presence of GLP-1R; GLP‐1R agonists ameliorated muscle weakness, muscle weight loss, and muscle inflammation through inhibiting muscle fiber necroptosis.
Sjoberg et al., 2014 [27]; Subaran et al., 2014 [34]; Wang et al., 2020 [28]	Experimental (humans)	GLP-1 infusion recruits skeletal muscle microvasculature.
Smits et al., 2015 [35]	Experimental (humans)	GLP-1 agonism through exenatide increases muscle microvasculature independent of nitric oxide.
Liu et al., 2016 [36]	Experimental (humans)	GLP-1 agonism through exenatide increases irisin, a myokine that is secreted in response to exercise playing a role in muscular benefits from exercise.
Abdulla et al., 2020 [25]	Experimental (humans)	GLP-1 agonism increases muscle protein synthesis in postprandial hyperaminoacidemic states.
Abdulla et al., 2023 [37]	Experimental (humans)	Among older adult males, femoral arterial GLP-1 infusion is associated with a significant rise in skeletal muscle microvascular blood flow in postprandial conditions.
Perna et al., 2016 [38]	Case series (humans)	GLP-1 agonism through liraglutide reduces fat mass and preserves muscle.
Ozeki et al., 2022 [26]	Pilot study (humans)	GLP-1 agonism through semaglutide maintains muscle mass while decreasing adiposity in patients who are obese and diagnosed with type II diabetes.

Discussion

In studies using mice models, biopsies showed the presence of GLP-1R [22,33]. Infusion of GLP-1R agonists led to muscle biopsy findings of decreased muscle atrophy [20,24,33] and inflammation [21,33] with additional observations of restored myofibrillar architecture [21,29], decreased intramyocellular lipid deposition [32], improvement in muscle vasculature [30,31], and increased preservation of mitochondria in muscle [22]. Human studies mirrored the above findings, including maintained or increased muscle mass [25,26,33,38], decreased adiposity [26,38], and increased muscle vasculature [35].

While the precise pathophysiology of the inflammatory myopathies is not fully understood, it is known to involve inflammation, atrophy, and necrosis of muscle tissue. In addition to regulating glucose homeostasis, GLP-1 agonists have been shown to suppress the expression of muscle atrophic factors, promote the effect of myogenic factors, and reduce inflammation and adiposity. There is increasing evidence to support the role of GLP-1R agonists in IIMs. These medications can serve as potentially promising therapies to supplement existing corticosteroid and immunosuppressant treatments for patients with IIM, helping alleviate some of the debilitating symptoms associated with the disease condition. Moreover, GLP-1R agonists may be helpful for individuals who have been unresponsive to current pharmacotherapy options as well as those who have concurrent metabolic syndrome and cardiovascular health concerns.

This scoping review has summarized key findings across research studies that discuss the potential therapeutic benefits of GLP-1R agonists for myositis. Considering the paucity of data-based literature on GLP-1R agonism in relation to inflammatory myopathy, this review provides an overview of current evidence in the field. However, there are some limitations to be noted. As articles were chosen based on perceived relevance, there is a possibility of selection and publication bias. Additionally, many of the articles included in the review were bench studies. There is a need for greater level-1, translational-based evidence, especially randomized clinical trials, to better assess the efficacy of GLP-1R agonists as a pharmacological treatment choice for patients with IIM. This includes understanding the specific mechanisms of action by which GLP-1R agonists can ameliorate and manage symptoms of the various IIM subtypes.

## Conclusions

Research studies involving both mouse models and human subjects have demonstrated that GLP-1R agonists contribute to decreased muscle atrophy; improved inflammation, adiposity, and vasculature; and mitochondrial preservation in muscle tissue. These findings suggest that GLP-1R agonists can play a therapeutic role for patients with IIM, who currently rely on a small range of medications for management. However, the existing published literature related to this field remains limited. As such, more research and funding for randomized controlled trials are needed to continue to assess the long-term benefits and potential side effects of GLP-1R agonists in IIM.
